# Estimation of Symmetric Channels for Discrete Cosine Transform Type-I Multicarrier Systems: A Compressed Sensing Approach

**DOI:** 10.1155/2015/151370

**Published:** 2015-10-18

**Authors:** María Elena Domínguez-Jiménez, David Luengo, Gabriela Sansigre-Vidal

**Affiliations:** ^1^ETSI Industriales, Universidad Politécnica de Madrid, C/José Gutiérrez Abascal 2, 28006 Madrid, Spain; ^2^ETSIS de Telecomunicación, Universidad Politécnica de Madrid, Carretera de Valencia Km 7, 28031 Madrid, Spain

## Abstract

The problem of channel estimation for multicarrier communications is addressed. We focus on systems employing the Discrete Cosine Transform Type-I (DCT1) even at both the transmitter and the receiver, presenting an algorithm which achieves an accurate estimation of symmetric channel filters using only a small number of training symbols. The solution is obtained by using either matrix inversion or compressed sensing algorithms. We provide the theoretical results which guarantee the validity of the proposed technique for the DCT1. Numerical simulations illustrate the good behaviour of the proposed algorithm.

## 1. Introduction

In wireless communications, the channel filter is usually time-varying; for this reason, it is necessary to estimate the channel filter from time to time. To this aim, some training symbols (i.e., symbols known both by the transmitter and by the receiver) are typically used. In this way, when the training symbols are transmitted by the channel, the received signal is used to extract the information about the channel filter. Some well-known techniques for channel estimation are based on the Discrete Fourier Transform (DFT); in this case, the training symbols are OFDM waveforms.

Additionally, if the channel filter is sparse (i.e., containing only a small amount of nonzero coefficients), then compressed sensing techniques can be applied. Compressed sensing (CS) algorithms approximate the sparsest solution to a linear system [1]. This is very useful when the solution depends on a small number of degrees of freedom and only a few measurements of the vector are observed. For this reason, in the last few years CS algorithms have been applied to a wide variety of scenarios in communications: cognitive radio, radar, antenna arrays, multicarrier communications, and so forth. When CS is applied to channel estimation problems, it is usually denoted as compressed channel sensing (CCS). Several CCS algorithms have been proposed in the literature for different types of channels arising in communication problems, such as ultrawideband channels, underwater acoustic communications, or multipath channels [2–5]. Most of these techniques are based on DFTs or spread spectrum signals.

In this work, we consider a multicarrier communications system that is based on the Discrete Cosine Transform Type-I (DCT1) even instead of the standard DFT. Some Discrete Cosine Transforms have been widely used in the context of multicarrier modulation (MCM), as an alternative to the DFT, due to their good properties (e.g., good performance under carrier frequency offset) [6–13]. In particular, in a very recent work [14] the DCT1 is applied for MCM communications. The main advantages of the DCT1 are as follows:

- The inverse of the DCT1 is the same transform DCT1, up to a scaling factor; so we can use the same transform at both the transmitter and the receiver [15].
- The convolution of two vectors is transformed by DCT1 into a pointwise product of their transforms (under some symmetry conditions on the vectors) [15, 16]. This is analogous to the circular convolution property of the DFT. This is a key property for signal reconstruction in MCM communications [14].

For these reasons, we investigate the use of DCT1 for channel estimation; in particular, we address the problem of estimation of whole-point symmetric (WS) channels by means of CS techniques in the DCT1 transform domain. The strategy consists of using only a few training symbols, which are transmitted through the channel, and reconstructing the impulse response of the filter in the receiver by using the same small number of measurements. Thus, the economy of the data can be exploited by CS algorithms, which are able to provide sparse filters.

In this work we will provide not only a new estimation procedure but also the training signals valid for our algorithm, and we will show that this technique is both simple and theoretically correct. These are the main contributions of this paper. Numerical simulations also illustrate the effectiveness of our results.

The paper is organized as follows. Firstly, in Section 2 we recall the general channel estimation problem. Secondly, in Section 3 the DCT1 is introduced and we obtain new important properties of this transform. Then, the proposed procedure is presented in Section 4, where its theoretical justification is also provided.  Section 6 contains some numerical examples that illustrate the behaviour of our algorithm. Finally, we highlight the main contributions of this work in Section 7.

## 2. The Channel Estimation Problem

**Figure 1:**
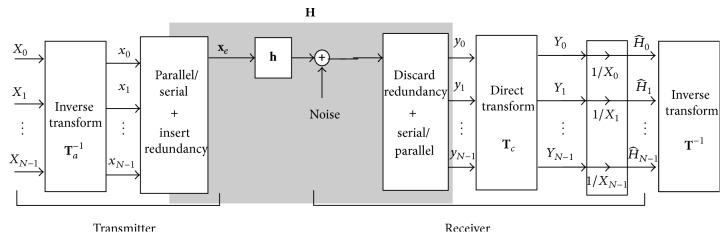
Block diagram of a multicarrier modulation communications system, including the channel estimation in the receiver.

Let us consider a multicarrier modulation communications system that performs an inverse transform **T**_*a*_^−1^ in the transmitter and a direct transform in the receiver **T**_*c*_, as shown in Figure 1. Let us consider also a channel with the following impulse response:

(1) $$\begin{array}{c} h={\left[\;h_{1-\nu },\ldots ,h_{-1},h_{0},h_{1},\ldots ,h_{\nu -1}\;\right]}^{⊤}. \end{array}$$

The transmission of an information symbol **x** = [*x*_0_,…, *x*_*N*−1_]^*⊤*^ through this channel results in a received symbol **y** = [*y*_0_,…, *x*_*N*+2*ν*−3_]^*⊤*^, such that

(2) $$\begin{array}{c} y_{k}=\overset{\nu -1}{\underset{m=1-\nu }{\sum }}h_{m}x_{k-m}+n_{k}, \end{array}$$

where *n*_*k*_ is a term related to the additive noise.

In multicarrier systems, in order to eliminate interblock interference, we often add to the original symbol **x** a left prefix **x**_lp_ and a right suffix **x**_rs_, both of length *ν* − 1:

(3) $$\begin{array}{c} x_{e}=\left[\begin{array}{c} x_{\text{lp}}\\ x\\ x_{\text{rs}} \end{array}\right]. \end{array}$$

In matrix form, the received data symbol **y** is given by

(4) $$\begin{array}{c} y=H\cdot x_{e}+n, \end{array}$$

where **H** is the Toeplitz matrix of size *N* × (*N* + 2*ν* − 2) defined by the filter:

(5) $$\begin{array}{c} \left[\begin{array}{cccccccc} h_{1-\nu } & \cdots & h_{0} & \cdots & h_{\nu -1} & 0 & \cdots & 0\\ 0 & h_{1-\nu } & \ddots & h_{0} & \ddots & h_{\nu -1} & \ddots & \vdots \\ \vdots & \ddots & \ddots & \ddots & \ddots & \ddots & \ddots & 0\\ 0 & \cdots & 0 & h_{1-\nu } & \cdots & h_{0} & \cdots & h_{\nu -1} \end{array}\right]. \end{array}$$

It is easy to see [8] that this received symbol can be written as

(6) $$\begin{array}{c} y=H_{\text{equiv}}\cdot x+n. \end{array}$$

Therefore, if we apply both the discrete transformations **T**_*a*_ and **T**_*c*_ in order to diagonalize **H**_equiv_,

(7) $$\begin{array}{c} T_{c}\cdot H_{\text{equiv}}\cdot T_{a}^{-1}=D, \end{array}$$

then

(8) $$\begin{array}{c} y=T_{c}^{-1}\cdot D\cdot T_{a}\cdot x+n, \end{array}$$

and denoting **Y** = **T**_*c*_ · **y**, **X** = **T**_*a*_ · **x** and **N** = **T**_*c*_ · **n**, we get

(9) $$\begin{array}{c} Y=D\cdot X+N. \end{array}$$

Now, the question is if we know the training symbol **x** and its corresponding received symbol **y**, is it possible to estimate **h**? The answer is* yes*, whenever there is an invertible **T** which transforms **h** into the elements of the diagonal matrix **D** = diag⁡(**H**_0_,…, **H**_*N*−1_). Hence, in the absence of noise, it suffices to compute **H**_*k*_ = **Y**_*k*_/**X**_*k*_; but in the presence of noise we only obtain an estimation in the transform domain:

(10) $$\begin{array}{c} {\hat{H}}_{k}=\frac{Y_{k}}{X_{k}}. \end{array}$$

Now we can recover the estimated filter as

(11) $$\begin{array}{c} \hat{h}=T^{-1}\cdot {\left[\;{\hat{H}}_{0},\ldots ,{\hat{H}}_{N-1}\;\right]}^{⊤}. \end{array}$$

Of course, this estimation would be exact in absence of noise. See Figure 1 for a general diagram of the channel estimation problem.

The existence of such transform **T** is a condition usually met in practice. For example, in OFDM systems the signal is extended by appending a cyclic prefix or suffix, so that the equivalent matrix **H**_equiv_ is circulant and diagonalized by the DFT transform. The diagonal matrix **D** contains the eigenvalues of **H**_equiv_, which in addition form the vector DFT(**h**). Hence, **h** is estimated simply by applying an inverse DFT.

As OFDM systems present poor behaviour under carrier frequency offset, other multicarrier modulation (MCM) techniques have been investigated, which are related to other transformations different from DFT. Among them, the eight types of Discrete Cosine Transforms (DCTs) have been studied in the literature, and for each one of them the corresponding extension technique has been proposed [6, 8, 9, 14]. These works focus on MCM signal reconstruction, and they provide good results due to the good properties of the DCTs. However, the channel estimation stage is essential in order to implement a DCT-MCM system in practice. For this reason, in this paper we apply the DCT1 to the channel estimation problem for the first time.

## 3. The Discrete Cosine Transform Type-I (DCT1) Even

The DCT1 even of an *N*-length signal is given by the matrix **C**_1*e*_, whose (*k*, *j*)th element is defined by

(12) $$\begin{array}{c} {\left(\;C_{1e}\;\right)}_{k,j}=a_{j}\cos\left(\;\frac{kj\pi }{N-1}\;\right),\;0\leq k,\;\;j\leq N-1, \end{array}$$

where

(13) $$\begin{array}{c} a_{j}=\left\{\begin{array}{cc} \frac{1}{\sqrt{2\left(N-1\right)}}, & \text{if }j=0,N-1,\\ \frac{2}{\sqrt{2\left(N-1\right)}}, & \text{otherwise}. \end{array}\right. \end{array}$$

This is the definition of **C**_1*e*_ given in [15], except for the normalization factor $\sqrt{2(N-1)}$; it has been introduced here in order to ensure the involution property, **C**_1*e*_^−1^ = **C**_1*e*_, which simplifies the numerical calculations. In this way, the direct and inverse DCT1 transforms are identical.

The first contribution of this work is the demonstration of the following theorem regarding the invertibility of some submatrices of the DCT1 matrix. This is a key property which guarantees that the channel filter can be obtained by means of a small amount of received data; this will be applied in the following section, when using compressed sensing techniques. Let us now state and prove this important property.

**Theorem 1 .**

Any *ν* × *ν* submatrix of **C**_1*e*_, whose columns have been extracted from the first *ν* columns of **C**_1*e*_, is invertible.

**Proof**

The submatrix formed by the first *ν* columns of **C**_1*e*_ is $C_{1e}\left[\begin{array}{c} I_{\nu }\\ O_{\left(N-\nu \right)\times \nu } \end{array}\right].$ Let us consider any *ν* × *ν* submatrix **B** of this matrix; our aim is to show that **B** is invertible. Notice that its *ν* rows can be indexed as 0 ≤ *k*_1_ < *k*_2_ < ⋯<*k*_*ν*_ ≤ *N* − 1, so the entries of **B** are

(14) bk,j=ajcos⁡πkjN−1,k∈k1,k2,…,kν,  j=0,…,ν−1.

To show that **B** is invertible, it suffices to prove that the unique vector **b** such that **B** **b** = 0 is **b** = 0. Let **b** = [*b*_0_, *b*_1_,…,*b*_*ν*−1_]^*⊤*^ be such vector; the condition **B** **b** = 0 is rewritten as

(15) $$\begin{array}{c} \overset{\nu -1}{\underset{j=0}{\sum }}a_{j}\cos\left(\;\frac{\pi k_{n}j}{N-1}\;\right)b_{j}=0,\;n=1,\ldots ,\nu . \end{array}$$

By defining *c*_*j*_ = *a*_*j*_ *b*_*j*_/2  (*j* = 0,…, *ν* − 1) we can rewrite the latter expression as

(16) $$\begin{array}{c} \overset{\nu -1}{\underset{j=0}{\sum }}2\cos\left(\;\frac{\pi k_{n}j}{N-1}\;\right)c_{j}=0,\;n=1,\ldots ,\nu . \end{array}$$

Our aim is to prove that the numbers *c*_*j*_ which fulfill (16) are necessarily null; it is equivalent to the fact that *b*_*j*_ = 0, *j* = 0,…, *ν* − 1, finishing the proof.

To this aim, let us now introduce the auxiliary self-reciprocal polynomial *q* of degree ≤2*ν* − 2:

(17) qz=cν−1+cν−2z+⋯+2c0zν−1+⋯+cν−2z2ν−3+cν−1z2ν−2.

Notice that, for any *z* ≠ 0, we have that

(18) $$\begin{array}{c} q\left(\;z\;\right)=z^{\nu -1}\overset{\nu -1}{\underset{j=0}{\sum }}\left(\;z^{j}+z^{-j}\;\right)c_{j}; \end{array}$$

thus, if *z* is a nonzero root of *q*, then also *z* ^−1^ is a root of *q*.

Our strategy is to prove that *q* has 2*ν* − 1 roots, say, more than its degree 2*ν* − 2; if this occurs, then *q* must be the null polynomial, and all its coefficients necessarily are 0, so *c*_*j*_ = 0, *j* = 0,…, *ν* − 1, and the claim follows. Let us find some roots of *q*:

(i) By denoting the complex numbers
  (19) $$\begin{array}{c} z_{n}=\exp\left(\;\frac{\pi k_{n}}{N-1}i\;\right),\;n=1,\ldots ,\nu , \end{array}$$
 it is easy to see that
  (20) $$\begin{array}{c} q\left(\;z_{n}\;\right)=z_{n}^{\nu -1}\overset{\nu -1}{\underset{j=0}{\sum }}2\cos\left(\;j\frac{\pi k_{n}}{N-1}\;\right)c_{j}=0, \end{array}$$
 where we have used (16). Hence, *z*_1_,…, *z*_*ν*_ are *ν* roots of *q*; note that there are *ν* different numbers because their arguments lie in [0, *π*] since
  (21) $$\begin{array}{c} 0\leq \frac{\pi k_{1}}{N-1}<\frac{\pi k_{2}}{N-1}<\cdots <\frac{\pi k_{\nu }}{N-1}\leq \pi . \end{array}$$
(ii) As already mentioned, for any *n* = 1,…, *ν*, also *z*_*n*_^−1^ is a root of *q*:
  (22) $$\begin{array}{c} z_{n}^{-1}=\exp\left(\;-\frac{\pi k_{n}}{N-1}i\;\right),\;n=1,\ldots ,\nu . \end{array}$$
 So there are *ν* different roots of *q* whose arguments lie in [−*π*, 0].
(iii) The union of the set of roots of (19) and (22) provide a total amount of 2*ν* different roots if *k*_1_ > 0 and *k*_*ν*_ < *N* − 1. In this case, *q* has more roots than its degree, so *q* is the null polynomial and the claim holds.
 In case *k*_1_ = 0, the corresponding root *z*_1_ = 1 has been counted twice; the same happens if *k*_*ν*_ = *N* − 1, because the root *z*_*ν*_ = −1 would appear twice. In these cases, we can only guarantee that there are 2*ν* − 2 different roots but it is easy to see that any self-reciprocal polynomial of even degree *q* satisfies the following property: if *z* = 1 (or *z* = −1) is a root of *q*, then it is a root of multiplicity at least 2. This implies that, in our case, *z*_1_ = 1 (or *z*_*ν*_ = −1) is a double root, so *q* has at least 2*ν* − 1 roots, concluding the proof.

## 4. Channel Estimation in DCT1 Multicarrier Systems

**Figure 2:**
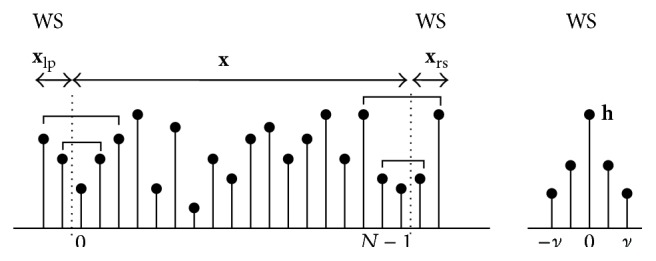
Symmetries in **x** and **h** to be used in DCT1-based systems (*N* ≫ (2*ν* + 1)) [14].

Let us assume that the channel filter presents whole-point (WS) symmetry: **h** = [*h*_*ν*−1_,…,*h*_1_, *h*_0_, *h*_1_,…,*h*_*ν*−1_]^*⊤*^. As we have already mentioned before, the interblock interference is eliminated by introducing as redundancy a left prefix **x**_lp_ and also a right suffix **x**_rs_, both of length *ν* − 1, into each data symbol to be transmitted. In order to apply DCT1, it is proved in [9, 14] that it suffices to consider the extended block **x**_*e*_ in (3) with the choice of prefix **x**_lp_ and suffix **x**_rs_ as follows:

(23) xlpn=xν−n,∀n=0,…,ν−2,xrsn=xN−2−n,∀n=0,…,ν−2

which means that we apply a whole-point symmetry (WS) on the left and on the right sides of the original symbol.  Figure 2 illustrates an example of the WS symmetric extension of **x**. The received vector (4) is then

(24) $$\begin{array}{c} y=H\cdot x_{e}+n=H_{\text{equiv}}\cdot x+n. \end{array}$$

It is proved in [14] that the corresponding **H**_equiv_ can be perfectly diagonalized via the DCT1:

(25) $$\begin{array}{c} C_{1e}\cdot H_{\text{equiv}}\cdot C_{1e}^{-1}=D, \end{array}$$

and the diagonal elements of **D** eigenvalues of **H**_equiv_ are themselves the DCT1 transform of the vector **h**_ZP_^*r*^:

(26) $$\begin{array}{c} H_{k}=\left(\;C_{1e}\cdot h_{\text{ZP}}^{r}\;\right)\left(\;k\;\right),\;k=0,\ldots ,N-1, \end{array}$$

where **h**_ZP_^*r*^ stands for the half-right filter of **h**, padded with zeroes:

(27) $$\begin{array}{c} h_{\text{ZP}}^{r}={\left[\;h_{0},\ldots ,h_{\nu -1},0,\ldots ,0\;\right]}^{⊤}. \end{array}$$

Thus, we have been able to find an easy solution to the channel estimation problem in DCT1 MCM communication systems. Following the general statement of the problem given in Section 2, we denote **Y**≔**C**_1*e*_ · **y**, **X**≔**C**_1*e*_ · **x**, and **N**≔**C**_1*e*_ · **n** and get the scheme

(28) $$\begin{array}{c} Y=D\cdot X+N, \end{array}$$

where **D** = diag⁡(**H**_0_,…, **H**_*N*−1_).

As the components of the training signal **X** are the symbols that can be stored in memory, from this equation we simply obtain an estimation of **H**_*k*_, for any component *k* such that **X**_*k*_ ≠ 0:

(29) $$\begin{array}{c} {\hat{H}}_{k}=\frac{Y_{k}}{X_{k}}. \end{array}$$

In other words, if all the 1-tap filters **X**_*k*_ are nonzero (*k* = 0,…, *N* − 1), then we compute the *N*-length vector $\hat{h}=C_{1e}^{-1}\cdot \hat{H}$, which gives a perfect estimation of **h**_ZP_^*r*^ in absence of noise, and we can straightforwardly obtain the symmetric channel filter **h** = [*h*_*ν*−1_,…, *h*_1_, *h*_0_, *h*_1_,…, *h*_*ν*−1_].

## 5. Compressed Channel Sensing for DCT1

Now, the question is what can we do if a component of **X** is null? In effect, this is a situation very common in practice. Indeed, we would like to have only a few training symbols **X**_*k*_ ≠ 0 and many null components **X**_*k*_ = 0. Moreover, how can we obtain an estimated vector **h**_ZP_^*r*^ = [*h*_0_,…, *h*_*ν*−1_, 0,…, 0] which is* sparse*? The answer to these two questions is given by two facts: on the one hand, we can apply compressed sensing techniques; on the other hand, the DCT1 matrix presents a key property which guarantees that the *ν* components of the sparse vector **h**_ZP_^*r*^ can be obtained by knowing only *ν* components of the vector ${\hat{H}}_{k}.$

Let us explain this idea in detail. If there are only *ν* nonzero symbols **X**_*k*_ ≠ 0, corresponding to the components *k* = *k*_1_,…, *k*_*ν*_, then only *ν* components of the vector $\hat{H}$ are defined, by means of (29). As **C**_1*e*_ · **h**_ZP_^*r*^ has length *N*, it is impossible to recover its *N* components, but at least we can try to estimate *ν* of them by means of the computed *ν* components of the estimated vector $\hat{H}$. Let us use the same notation $\hat{H}$ to define the *ν*-length vector which contains the *ν* known components of  (29). Then, we need to find the sparse vector **h**_ZP_^*r*^ that minimizes the norm

(30) $$\begin{array}{c} \min\left\|\;C\cdot h_{\text{ZP}}^{r}-\hat{H}\;\right\|, \end{array}$$

where **C** denotes the submatrix of **C**_1*e*_ formed by its *ν* corresponding rows *k* = *k*_1_,…, *k*_*ν*_.

Besides, we can exploit the structure of **h**_ZP_^*r*^, which has *N* − *ν* final zeroes, so we can write

(31) $$\begin{array}{c} C\cdot h_{\text{ZP}}^{r}=C\cdot \left[\begin{array}{c} h^{r}\\ 0 \end{array}\right]=C\cdot \left[\begin{array}{c} I_{\nu }\\ O_{\left(N-\nu \right)\times \nu } \end{array}\right]\cdot h^{r}=C_{f}\cdot h^{r}, \end{array}$$

where we have denoted **h** ^*r*^ = [*h*_0_,…, *h*_*ν*−1_]^*⊤*^,

(32) $$\begin{array}{c} C_{f}=C\cdot \left[\begin{array}{c} I_{\nu }\\ O_{\left(N-\nu \right)\times \nu } \end{array}\right], \end{array}$$

and **C**_*f*_ stands for the *ν* × *ν* submatrix of **C**_1*e*_ containing the* first ν* columns of **C**_1*e*_ and the corresponding rows *k* = *k*_1_,…, *k*_*ν*_. In this way, we have the following minimization problem:

(33) $$\begin{array}{c} \min\left\|\;C_{f}\cdot h^{r}-\hat{H}\;\right\|, \end{array}$$

where vectors **h** ^*r*^ and $\hat{H}$ have length *ν*.

Compressed sensing techniques show that it is possible to achieve the sparsest vector **h** ^*r*^ if its sparsity order is *s* < spark(**C**_*f*_)/2 (the spark of a square matrix is its rank plus 1). As we have proved in Section 3, our Theorem 1 guarantees that the rank of any square submatrix of the first columns of **C**_1*e*_ is maximum, so **C**_*f*_ has maximum rank. This means that we can reconstruct sparse filters **h** ^*r*^ of sparsity order *s* < (*ν* + 1)/2. In practice, it is possible to recover vector **h** ^*r*^ in two ways:

(i) As **C**_*f*_ is invertible, we can simply define $h^{r}=C_{f}^{-1}\cdot \hat{H}$ so as to get null error ($C_{f}\cdot h^{r}-\hat{H}=0).$ This is true in absence of noise, but the drawback in practice is that we may obtain a nonsparse vector **h** ^*r*^.
(ii) Alternatively, as **C**_*f*_ has maximum rank *ν*, we can apply some well-known algorithms used in CS scenarios (e.g., Lasso techniques, OMP, and CoSamp [5]) in order to find the sparse solution **h** ^*r*^ of the problem. These algorithms converge when the matrix of the linear system **C**_*f*_ satisfies either the Restricted Isometry Property (RIP) or a weaker property regarding the* coherence* of **C**_*f*_. Luckily, we can also guarantee that this good property is fulfilled by the DCT1, by placing *s* symbols at equally spaced positions. This procedure has been applied in our simulations, and this clear advantage is the reason why the DCT1 matrix performs well for compressed sensing techniques in the simulations.

*Summary of the procedure is as follows*:

(1) Choose a training signal **X** of length *N*, and compute **C**_1*e*_^−1^ · **X** = **x**.
(2) Apply a whole-point symmetry of length *ν* − 1 at both edges of **x** so as to obtain **x**_*e*_ of length *N* + 2*ν* − 2.
(3) Transmit **x**_*e*_ through the channel.
(4) Take the *N* central components of the received vector, which form **y**.
(5) Apply the DCT1 block: **Y** = **C**_1*e*_ · **y**.
(6) Compute ${\hat{H}}_{k}=Y_{k}/X_{k}$.
(7) In case **X**_*k*_ ≠ 0 for all *k*, obtain $C_{1e}^{-1}\cdot \hat{H}$ which is the desired estimation of the half-right filter **h**_ZP_^*r*^ = [**h** ^*r*^, 0,…, 0]^*⊤*^.
(8) In case some components of **X**_*k*_ are null, and at least *ν* components of **X**_*k*_ are nonzero, find the solution **h** ^*r*^ of the problem
  (34) $$\begin{array}{c} \min\left\|\;C_{f}\cdot h^{r}-\hat{H}\;\right\| \end{array}$$
 that can be solved via CS techniques or simply defining
  (35) $$\begin{array}{c} h^{r}=C_{f}^{-1}\cdot \hat{H}. \end{array}$$
(9) In any case, from **h** ^*r*^ = [*h*_0_,…, *h*_*ν*−1_] by WS symmetry we get the estimated filter channel
  (36) $$\begin{array}{c} h=\left[\;h_{\nu -1},\ldots ,h_{1},h_{0},h_{1},\ldots ,h_{\nu -1}\;\right]. \end{array}$$

## 6. Numerical Results

In this section, we analyse the behaviour of the proposed compressed channel sensing (CCS) scheme by testing it on three channels: a fixed simple channel of length *L* = 7 (i.e., *ν* = 4), a more challenging fixed nonminimum phase channel of length *L* = 11 (i.e., *ν* = 6), and a perturbed symmetric version of the ITU-T M.1225 pedestrian channel A [17]. In all cases, a sparse signal is constructed in the DCT1 domain by setting *X*_*k*_ = 1 if *k* = *rP* (for *P* = (*N* − 1)/(*ν* − 1)) and *X*_*k*_ = 0 otherwise. Hence, *X*_*k*_ is a *ν*-sparse vector containing only *ν* nonnull elements uniformly distributed, as stated in the previous section. For instance, when *L* = 7 we have *P* = 85 and the nonnull elements are only *X*_0_, *X*_85_, *X*_170_, and *X*_255_, whereas for *L* = 11 we have *P* = 51 and the nonnull elements are *X*_0_, *X*_51_, *X*_102_, *X*_153_, *X*_204_, and *X*_255_. In this way, we are truly performing a compressed sensing of the channel, since we are only exploring certain elements (which correspond to particular frequencies) in the transformed domain.

The inverse DCT1 is then performed and the time-domain transmitted vector **x** is passed through the filter with symmetric impulse response **h**. Then, zero-mean additive white Gaussian noise (AWGN) with variance *σ*_*w*_^2^ is added, obtaining the received vector **y**. The length *N* DCT1 of the *N* central elements of this vector is now computed, resulting in **Y**. Finally, the *ν* elements of **Y** corresponding to the nonnull positions of **X** are extracted and a length *ν* inverse DCT1 is performed on them to estimate the right-half of the channel's impulse response. The rest of the channel is reconstructed exploiting its symmetry. The performance measure used is the reconstruction signal to noise ratio (SNR),

(37) $$\begin{array}{c} \hat{\text{SNR}}\left(\;\text{dB}\;\right)=10\;\log_{10}\frac{P_{e}}{P_{h}}, \end{array}$$

where *P*_*h*_ = (1/*L*)**h** ^*⊤*^ **h**, *P*_*e*_ = (1/*L*)(**h** − **h** ^*r*^)^*⊤*^(**h** − **h** ^*r*^), and *L* is the channel's length.

As a first example, we select the following *L* = 7 channel:

(38) $$\begin{array}{c} h={\left[\;0.05,0.25,-0.5,1,-0.5,0.25,0.05\;\right]}^{⊤}. \end{array}$$

We set the length of the DCT1 to *N* = 256 and check the behaviour of the CCS scheme as the channel's SNR increases from −10 dB to 30 dB using only 4 training pilots. *N*_*s*_ = 2000 simulations are performed for each SNR.

**Figure 3:**
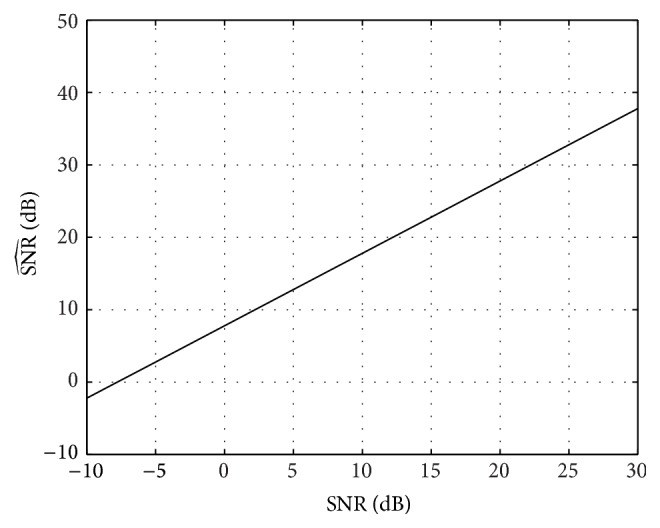
Channel reconstruction SNR ($\hat{SNR}(dB)$) as a function of the signal power to noise ratio (SNR(dB)). The length of the channel is *L* = 7, the length of the DCT1 is *N* = 256, and *N*_*s*_ = 2000 simulations have been performed.

Figure 3, which displays the reconstruction SNR as a function of the channel's SNR, shows that an increasingly accurate estimation of the channel can be obtained as the SNR increases, even by using only *N*_*p*_ = 4 pilots. It can be seen that the reconstruction SNR increases linearly as the signal power to noise ratio increases. Indeed, the following relationship can be established:

(39) $$\begin{array}{c} \hat{\text{SNR}}\left(\;\text{dB}\;\right)=\text{SNR}\left(\;\text{dB}\;\right)+\Delta \text{SNR}\left(\;\text{dB}\;\right), \end{array}$$

where SNR(dB) = 10log_10_(*P*_*x*_/*σ*_*w*_^2^), with *P*_*x*_ = (1/*N*)**x** ^*⊤*^ **x**, and ΔSNR(dB) = 7.78 in this case. This shows that an increase in SNR of 7.78 dB in the reconstruction is obtained with respect to the channel's SNR.

**Figure 4:**
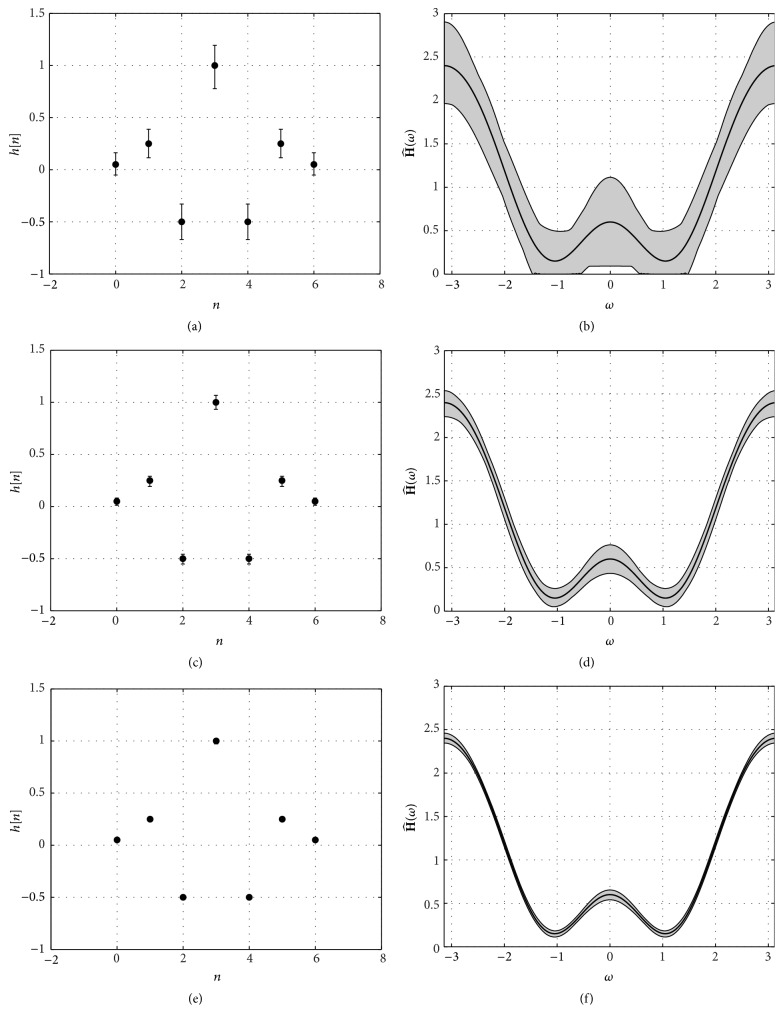
(a), (c), and (e) Estimated channel's impulse response for SNR = 0 dB, SNR = 10 dB, and SNR = 20 dB, respectively. (b), (d), and (f) Estimated channel's frequency response for SNR = 0 dB, SNR = 10 dB, and SNR = 20 dB, respectively. In all cases the length of the channel is *L* = 7, the length of the DCT1 is *N* = 256, and *N*_*p*_ = 4 pilot subcarriers are used in the transmitter.

Figure 4 shows three examples of the estimated channel's impulse and frequency responses for three signal to noise ratios: SNR = 0 dB, SNR = 10 dB, and SNR = 20 dB. The channel's impulse response is displayed on the left hand side (central dot with the true values and bar spanning the range between the minimum and maximum recovered values), whereas the right hand side shows the channel's frequency response (true value in black line and shaded area showing the range between maximum and minimum values). Note the substantial decrease in the variation of the coefficients of the channel's impulse response as the SNR increases (indeed, for SNR = 20 dB the bars cannot be appreciated, since the recovered coefficients are always virtually identical to the true coefficients) and the corresponding improvement in the estimation of the channel's frequency response (with a decrease in the shaded area).

**Figure 5:**
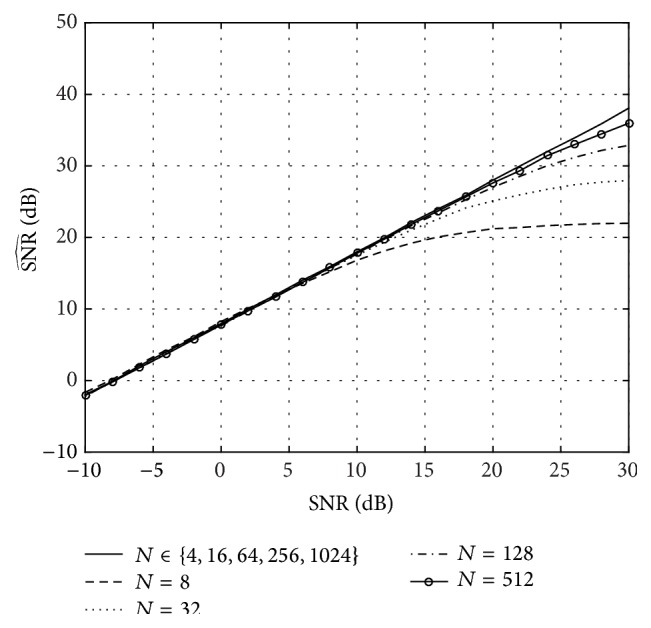
Channel reconstruction SNR ($\hat{SNR}(dB)$) as a function of the signal power to noise ratio (SNR(dB)) for different values of *N*. The length of the channel is *L* = 7 and *N*_*s*_ = 2000 simulations have been performed.

We have also tested the effect of the number of subcarriers, *N*, by using *N* = 2^*n*^ for *n* = 2,3,…, 10 (i.e., *N* = 4,8,…, 1024). The result, displayed in Figure 5, shows that the value of *N* is irrelevant (in terms of accuracy of the reconstructed channel), as long as *P* = (*N* − 1)/(*ν* − 1) is an integer number and the pilot carriers can be uniformly distributed (as it happens in this case for *N* ∈ {4,16,64,256,1024}). When *P* is not an integer number, the pilots cannot be uniformly distributed and an approximation error is obtained (as seen in the cases *N* = 8, *N* = 32, *N* = 128, and *N* = 512). However, this error decreases as *N* increases and can be completely avoided by zero-padding the channel's impulse response until *P* is integer.

As a second example, we consider a length *L* = 11 non-minimum-phase channel:

(40) h=0.9801,−0.5600,0.4799,0.7472,−0.2728,1,−0.2728,0.7472,0.4799,−0.5600,0.9801⊤.

**Figure 6:**
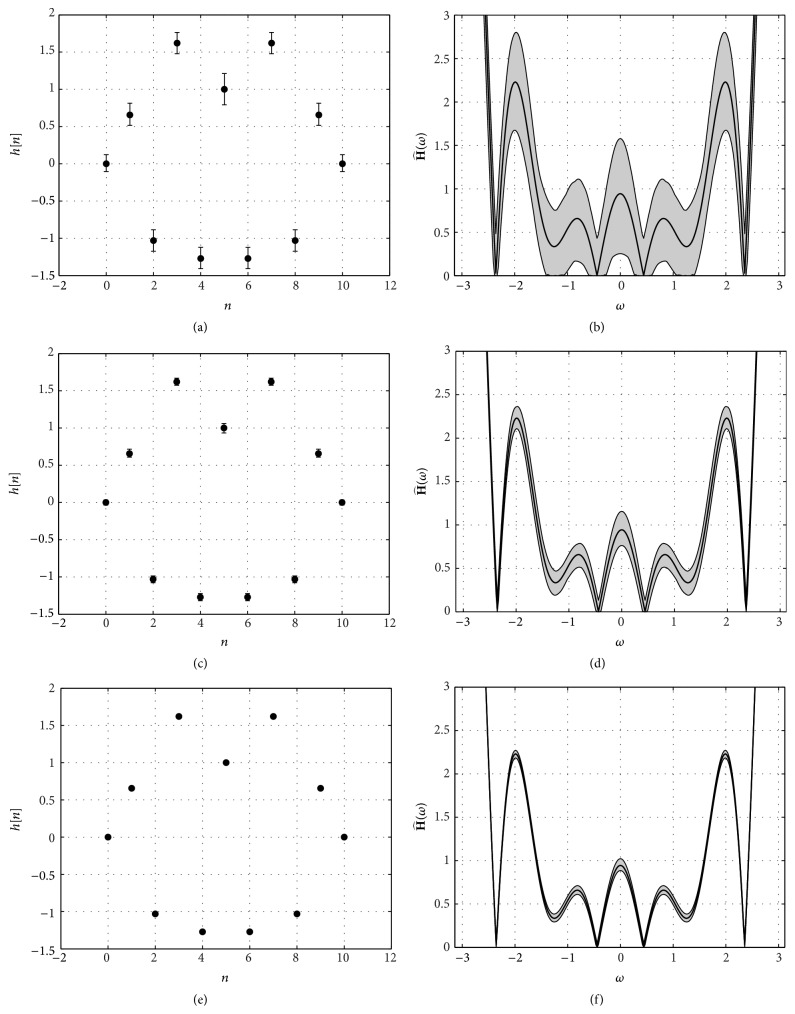
(a), (c), and (e) Estimated channel's impulse response for SNR = 0 dB, SNR = 10 dB, and SNR = 20 dB, respectively. (b), (d), and (f) Estimated channel's frequency response for SNR = 0 dB, SNR = 10 dB, and SNR = 20 dB, respectively. In all cases the length of the channel is *L* = 11, the length of the DCT1 is *N* = 256, and *N*_*p*_ = 4 pilot subcarriers are used in the transmitter.

We set again the length of the DCT1 to *N* = 256 and check the behaviour of the CCS scheme as the channel's SNR increases from −10 dB to 30 dB using only 6 training pilots. *N*_*s*_ = 2000 simulations are performed for each SNR. However, even though this channel is much more challenging than the previous one, similar results are obtained in terms of the reconstruction error. Indeed, (39) is also valid in this case and the reconstruction SNR versus channel's SNR curve for this channel (not shown) is virtually identical to Figure 3. In fact, we have also tested several other (both minimum and nonminimum phase) channels and this result seems to apply to all of them.  Figure 6 shows three examples of the estimated channel's impulse and frequency responses for three signal to noise ratios: SNR = 0 dB, SNR = 10 dB, and SNR = 20 dB.

**Figure 7:**
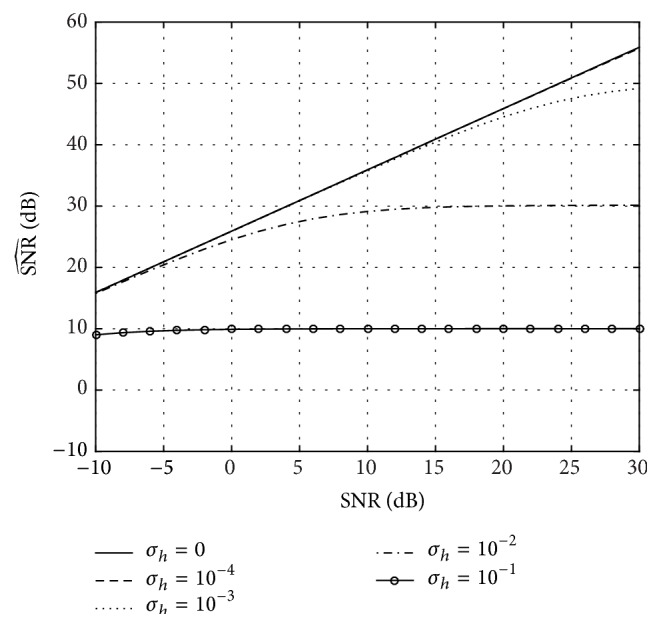
Channel reconstruction SNR ($\hat{SNR}(dB)$) as a function of the noise variance in the coefficients of *h*[*n*] (*σ*_*h*_^2^) for *N* = 4096. The length of the channel is *L* = 391 and *N*_*s*_ = 2000 simulations have been performed.

Finally, we test our approach on a perturbed symmetric version of the ITU-T M.1225 pedestrian channel A. The pedestrian channel A was generated using Matlab's  stdchan  function using a carrier frequency *f*_*c*_ = 2 GHz, a sampling period *T*_*s*_ = 10 ns, and a length *L*_0_ = 196. The resulting channel's impulse response, **h**_0_ = [*h*_0_[0],…, *h*_0_[*L*_0_ − 1]], is very sparse, since it typically has only 3 nonnull coefficients. The symmetric channel's impulse response is constructed as **h** = [**h**_0_[*L*_0_ − 1 : −1 : 1], **h**_0_], so its length is *L* = 2*L*_0_ − 1 = 391 and we have *P* = 21. Then, the coefficients of *h*[*n*] are perturbed by adding independent white Gaussian noise samples with variance *σ*_*h*_^2^ to each of them in order to analyze the effect of the lack of symmetry, typical of real-world channels. The results are shown in Figure 7: a small lack of symmetry (e.g., *σ*_*h*_^2^ = 10^−4^ or *σ*_*h*_^2^ = 10^−3^) practically has no effect; a moderate amount (e.g., *σ*_*h*_^2^ = 10^−2^) lowers the performance but still provides a good estimate of the channel (with a reconstruction SNR around 30 dB); a large lack of symmetry (e.g., *σ*_*h*_^2^ = 10^−1^) results in a low reconstruction SNR (around 10 dB), as the reconstructed channel is approximately equal to the symmetric part of the true channel. This highlights the limitations of our approach but also its potential in many approximately symmetric real-world channels (e.g., channels with a large central coefficient and small not completely symmetric coefficients around it).

## 7. Conclusions

In this work, we have presented a general procedure for the estimation of any symmetric channel filter for multicarrier communication systems based on the Discrete Cosine Transform Type-I (DCT1) even. For any training signal transmitted through the channel, at the receiver, we show how to take into account the information of the training symbol so as to estimate the channel filter. The main contribution of this work is that it is possible to estimate the channel filter with a small amount of training signals, just knowing a small amount of the received samples, and regardless of the location of these samples. This is an important consequence of the good properties of the DCT1 matrix that have been also proved here for the first time. Thus, our proposed procedure with the DCT1 formulation meets the conditions that guarantee perfect estimation of the channel filter in absence of noise, whereas in noisy scenarios a very good estimation can also be achieved. We have also designed specific sparse training signals for our DCT1 procedure and showed that it can also be applied to channels whose impulse response is only approximately symmetric with good results. Future research lines include extending these procedure to nonsymmetric channels.
